# Cardiovascular Impact of Androgen Deprivation Therapy: from Basic Biology to Clinical Practice

**DOI:** 10.1007/s11912-023-01424-2

**Published:** 2023-06-05

**Authors:** Janice Kim, Kendall Freeman, Alyssa Ayala, McKay Mullen, Zijie Sun, June-Wha Rhee

**Affiliations:** 1 Department of Medicine, City of Hope Comprehensive Cancer Center, Duarte, CA 91010 USA; 2 Stanford Cardiovascular Institute, Stanford, CA 94305 USA; 3 Department of Cancer Biology and Molecular Medicine, City of Hope Comprehensive Cancer Center, Duarte, CA 91010 USA

**Keywords:** Prostate cancer, Testosterone, Androgen deprivation therapy, Cardiovascular complications

## Abstract

**Purpose of the Review:**

There have been increasing reports of cardiovascular complications of androgen deprivation therapy (ADT) leading to worse outcomes among patients with prostate cancer. While this may result from the direct effects of androgen suppression in the cardiovascular systems, there are ADT-type-specific distinct cardiovascular complications suggestive of mechanisms beyond androgen-mediated. Thus, it is critical to understand the biological and clinical impact of ADT on the cardiovascular system.

**Recent Findings:**

Gonadotropin-releasing hormone (GnRH) agonists cause increased cardiovascular events compared to GnRH antagonists. Androgen receptor antagonists are linked to an increased risk of long QT syndrome, torsades de pointes, and sudden cardiac death. Androgen synthesis inhibitors are associated with increased rates of hypertension, atrial tachyarrhythmia, and, in rare incidences, heart failure.

**Summary:**

ADT increases the risk of cardiovascular disease. The risk among ADT drugs differs and must be evaluated to develop a medically optimal plan for prostate cancer patients.

## Introduction

ADT is used to lower androgen levels to castration levels through surgical or pharmacologic therapies and has emerged as one of the leading therapies for prostate cancer. While cancer-related survival has significantly improved following the use of ADT, there have been increasing reports of cardiovascular (CV) complications associated with ADT [1]. Recent studies have documented a broad spectrum of cardiovascular complications associated with ADT, including but not limited to stroke, myocardial infarction, QT prolongation, arrhythmia, heart failure, and hypertension [2], suggesting significant cardiovascular complications following the use of ADT. Herein, we will first review the biology underlying the cardiovascular impact of androgens. We will then review the types of ADT review the types of ADT used in clinical practice, evidence of cardiovascular complications associated with each type, and proposed biological mechanisms underlying these cardiovascular effects. Finally, we will review CV management considerations for patients being treated with ADT, based on current evidence and guidelines.

## Biology of Androgen Signaling

**Fig. 1:**
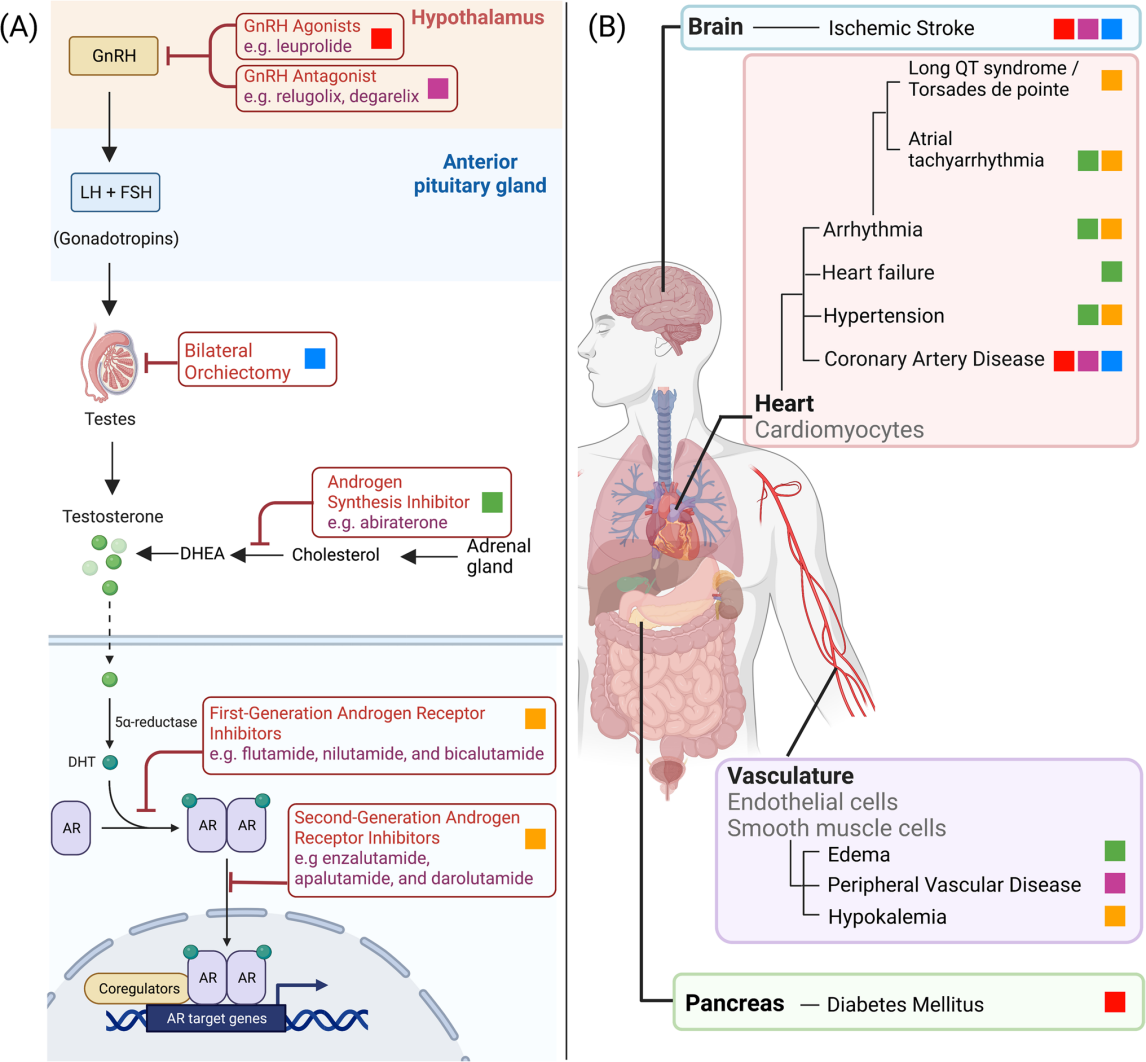
Mechanism of actions and cardiovascular consequences of androgen deprivation therapies. **A** Mechanisms of actions of various types of androgen deprivation therapies (ADT). **B** Adverse cardiovascular effects of ADTs. The colored boxes denote individual types of ADT; red, GnRH agonists; purple, GnRH antagonists; blue, bilateral orchiectomy; green, androgen synthesis inhibitor; orange, androgen receptor inhibitors. Created with Biorender.com

Androgens are male sex hormones involved in the development and maintenance of reproductive tissues (Fig. 1A) [3]. In males, the two predominant androgens are testosterone and dihydrotestosterone (DHT) [3]. Testosterone synthesis is regulated through the hypothalamic-pituitary–gonadal (HPG) axis [4]. The process begins in the hypothalamus with the secretion of gonadotropin-releasing hormone. Luteinizing hormone (LH) and follicle-stimulating hormone (FSH) are then released from the pituitary [5] and travel through the bloodstream to the testes [6]. LH binds to the receptors in the Leydig cells, which promote the production of testosterone [5]. Lastly, testosterone can be further processed to its more potent form (DH) via the enzyme 5-α reductase [5, 7].

Androgen signaling involves an androgen receptor (AR) and its ligand, androgen. AR is encoded on an X chromosome [7] and is responsible for mediating the targeted biological effects of androgens [3]. AR is composed of a nuclear transcription factor and a steroid hormone receptor region [5]. When androgen is bound to the receptor region, the AR undergoes conformational changes and migrates to the nucleus of the cell [5]. Once in the nucleus, the AR induces a series of downstream gene transcriptions, leading to the targeted effects of androgens [3] in both concentration and tissue-specific manners [4]. Androgen action is vital for diverse functions in many biological processes throughout the body, including including reproductive, musculoskeletal, cardiovascular, and hematopoietic systems.

## The Role of Androgens in the Cardiovascular System

The role of androgens in the CV system has been actively studied throughout the past few decades. Historically, testosterone, the main form of androgens, was believed to increase the risk for cardiovascular disease (CVD) [8]; men were found to be twice as likely as women to experience CVD. However, this sex discrepancy becomes insignificant when women undergo menopause, and it is now largely accepted that the sex discrepancy is largely driven by the cardioprotective role of estrogen during pre-menopause. Nevertheless, the cardiovascular effects of testosterone remain rather controversial. Some retrospective studies show that men receiving supplemental testosterone have a higher risk of CV events [9], suggesting that excessive testosterone may indeed accelerate CVD. However, deficient or low endogenous testosterone has also been associated with increased risk of CVD with an unfavorable metabolic profile and negative cardiovascular impact [4, 10], indicating complex and potentially bimodal effects of testosterone in the cardiovascular system. Therefore, in this section, we will systematically review the current evidence highlighting the effects of testosterone on CV health.

While still in dispute, an increasing body of literature has highlighted the protective effects of testosterone on the CV system (Table 1). *Vascular impact*: Testosterone plays key roles in various physiological processes, many of which are potentially linked to reduced CV risk and cardioprotection. It has been well documented that endothelial progenitor cells (EPCs) are crucial in promoting endothelial healing and maintaining vascular integrity [11]. Studies have shown that testosterone modulates vascular endothelial cell growth by acting on endothelial cell growth by acting on EPCs, thereby inducing vasorelaxation by directly enhancing nitric oxide synthesis [12]. Other androgens, such as dehydroepiandrostenedione (DHEA), have also been found to stimulate endothelial cell proliferation and angiogenesis [13]. Additionally, studies suggest DHT may regulate endothelial function by controlling inflammatory responses mediated by nuclear factor kappa B [14]. Overall, testosterone’s role in vascular homeostasis and vasomotor tone suggests favorable effects of androgens in the vasculature. *Myocardial impact*: Testosterone has also been shown to augment the beneficial effects of alpha1-adrenergic receptor stimulation in the heart, which conferred cardioprotection in patients with ischemic heart disease [13]. Cardioprotective effects included reduced myocardial injury and arrhythmias, as well as improved contractile recovery following ischemic and reperfusion injury. These effects were abolished or attenuated with androgen receptor blockade, indicating an androgen receptor-mediated mechanism against ischemic injury [13]. Other studies have reported additional mechanisms through which testosterone modulates cardioprotective signaling. Short-term stimulation of cardiomyocytes with testosterone increases AMP-activated protein kinase (AMPK), an important cardiomyocyte regulator with beneficiary cardiometabolic and anti-hypertrophic effects [15]. Testosterone also improves mitochondrial function, insulin sensitivity, and glucose uptake in cardiac cells, all of which may contribute to improved myocardial energetics in both the healthy and injured state [16]. Additional beneficiary myocardial effects of androgens have been suggested with various animal models. When rodents were subjected to bilateral gonadectomy, the density of 1,4-dihydropyridine (DHP) receptors (L-type Ca^2+^ channels) in the hearts was significantly reduced. This effect was reversed by testosterone replacement, suggesting that testosterone has important effects on cardiac contractility by increasing inward Ca^2+^ currents [17]. Gonadectomy has also been shown to increase action potential duration in individual myocytes, which may trigger myocardial arrhythmia [18]. Together, these findings support the notion that testosterone has an important role in regulating the cardiac action potential and Ca^2+^ homeostasis, both of which have important implications in CV health.

**Table 1:** Effects of androgens on the cardiovascular system

First author, Year	Primary CV endpoint	Findings
Beneficial effects of endogenous testosterone		
Dockery 2003	Vascular function	Free testosterone was associated with increased systemic arterial compliance (*r* = 0.507; *p* < 0.001)
Deenadayalu 2012		Testosterone induces coronary artery relaxation by activating calcium-activated potassium channels
Campelo 2012		Testosterone directly acts on endothelial nitric oxide production to modulate endothelial cell growth and platelet aggregation
Li 2014		Dihydrotestosterone increases endothelial progenitor cell proliferation and adhesive ability, promoting cardiovascular health
Norata 2006		By inhibiting the expression of inflammatory molecules (e.g., IL-6, Cox-2, and PAI-1), dihydrotestosterone may positively modulate endothelial function
Callies 2003	Myocardial protection	Testosterone administration improves recovery from myocardial ischemia
Malkin 2004		Hypogonadal men receiving testosterone replacement therapy had delayed time to ischemia, which was assessed by time to 1 mm ST depression (*p* = 0.0002)
Tsang 2008		Testosterone stimulates cardiac α1-adrenoceptors, reducing myocardial injury induced by ischemia and noradrenaline
Dobrzycki 2003		Compared to patients with higher levels of free-testosterone (86.10 pmo/l), patients with low free-testosterone levels (23.85 pmol/l) had significantly lower ejection fraction (51.85 vs. 61.30)
Agledahl 2009	Cardiovascular events	Elderly men with low testosterone had faster tissue-induced coagulation (5.1 ± 1.0 min vs. 5.7 ± 1.3, *p* = 0.039), suggesting that physiological levels of testosterone has anti-thrombotic properties
Ohlsson 2011		Elderly men with high serum testosterone (> 550 ng/dl) had a reduced 5-year risk of CV events than those with lower serum testosterone (HR, 0.70; 95% CI), even after adjusting for traditional CV risk factors
Effects of supplemental testosterone		
Baillargeon 2014	Beneficial effect on the CV system	Testosterone therapy was modestly protective against MI in men with high MI risk (HR, 0.69; 95% CI, 0.53–0.92). There was no increased risk for MI in men treated with intramuscular testosterone (HR, 0.84; 95% CI, 0.69–1.02)
Cheetham 2017		Testosterone prescriptions among men with androgen deficiency were associated with decreased risk of stroke events (stroke and TIA) (HR, 0.72; 95% CI, 0.62–0.84) and combined cardiac events (acute MI, SCD, UA, and revascularization procedures) (HR, 0.66; 95% CI, 0.60–0.72)
Crisostomo 2006	Adverse effect on the CV system	In an animal study, postischemic recovery of LVDP was significantly higher in females (71.7 ± 3.3%) and castrated males (64.5 ± 6.0%) than in females and castrated males who received acute testosterone infusion (26.1 ± 7.5%; 7.1 ± 1.3%), respectively
Xu 2013		Exogenous testosterone increased the risk of a cardiovascular-related event (OR, 1.54; 95% CI 1.09–2.18)
Vigen 2013		Testosterone replacement therapy was associated with increased risk of mortality, myocardial infarction, and stroke, regardless of presence of preexisting coronary artery disease (HR, 1.29; 95% CI, 1.04 to 1.58)
Gagliano-Jucá 2020		Testosterone administration was associated with increases in leukocyte and platelet counts (*p* < 0.001), both of which have known associations with cardiovascular and thromboembolic risks
Effects of testosterone deficiency		
Shores 2006	Adverse effect on the CV system	Male veterans with low testosterone levels had an increased mortality rate compared to those with equivocal or normal testosterone levels
Laughlin 2008		Low free testosterone levels in older men increased the risk of cardiovascular and respiratory related mortality (HR, 2.29; 95% CI, 1.25–4.20)
Vlachopoulos 2013		Subjects in the lowest total testosterone tertile (< 4.0 ng/ml) had an increased the risk for MACE compared to those in the highest TT tertile (> 4.9 ng/ml) after adjusting for age, systolic blood pressure, and risk factors. (all *p* < 0.05). MACE included CV death, stroke, TIA, CAD, and PAD
Babcock 2022		Brachial artery flow-mediated dilation was reduced in middle-aged and older men with lower testosterone compared to those with higher testosterone (*p* = 0.021). Low testosterone levels may contribute to accelerated vascular aging, which may increase risk for age-associated cardiovascular disease

Abbreviation: *CAD*, coronary artery disease; *CI*, confidence interval; *CV*, cardiovascular; *CVM*, cardiovascular mortality; *CrVD*, cerebrovascular death; *DM*, diabetes mellitus; *HF*, heart failure; *HR*, hazard ratio; *LVDP*, left ventricular diastolic pressure; *MACE*, major adverse cardiovascular event; *MI*, myocardial infarction; *PAD*, peripheral artery disease; *SCD*, sudden cardiac death; *TIA*, transient ischemic attack; *TT*, total testosterone; *UA*, unstable angina

Taken together, it can be concluded that physiological levels of androgens have beneficiary/protective effects on the CV system. Following this line of thought, it can be postulated that low levels of endogenous testosterone may lead to increased risk for adverse CV events. In cases of male hypogonadism, a condition of testosterone deficiency (TD), current data indicate a close relationship with the development of various cardiovascular risk factors, such as hyperlipidemia [8]. It is also well-established that TD is associated with increased adiposity, insulin resistance (IR), type 2 diabetes mellitus, hypertension, atherosclerosis, CVD, and incidence of mortality [19]. However, whether testosterone replacement therapy (TRT) provides benefits in patients with hypogonadism remains elusive. While some observational studies report beneficiary effects of TRT [20], other studies indicate no significant benefit or possible increased risk. This discrepancy may be due, in part, to the overall doses and resultant serum testosterone concentrations, such as high peak concentration. Thus, while total endogenous testosterone levels appear to be inversely associated with the risk of CV events, highlighting the cardioprotective effects, [22] the optimal strategy for replacing serum testosterone is to be further elucidated.

## Types of Androgen Deprivation Therapy

Prostate cancer (PCa) is primarily considered androgen-dependent with its proliferation stimulated by testosterone and DHT signaling [1, 23], although in a minor subset of “castration-resistant” PCa, cancer proliferation can become resistant or less sensitive to the androgen effects. Since androgens are critical in PCa growth, ADT has been a cornerstone therapy to slow proliferation and even cause regression of cancer cells. The goal of ADT is to suppress testosterone levels to castration levels through surgical or pharmacological castration [2]. In this section, we will review the four main types of ADT. These ADTs exert their effects through differing mechanisms by focusing on specific aspects of the HPG axis and testosterone synthesis, ultimately leading to a castration level of serum testosterone. While ADTs have led to the regression of PCa in most patient cases, significantly improving their cancer outcomes, unforeseen cardiovascular side effects have surfaced. Studies have demonstrated varying severity of cardiovascular complications associated with specific ADTs, which may be related to the specific mechanisms of each ADT type. Therefore, in the following section, we will focus on the types and mechanisms of the commonly used ADTs.

### Gonadotropin-Releasing Hormone Agonists and Antagonists

The most commonly used form of ADT is gonadotropin-releasing hormone (GnRH) modulators: GnRH agonists (e.g., leuprolide) and GnRH antagonists (e.g., relugolix, degarelix). GnRH modulators exert their effects on the signaling between the hypothalamus and pituitary of the HPG axis. The primary mechanism of GnRH agonists is the overstimulation of GnRH receptors, followed by subsequent desensitization [24]. Initially, overstimulation of GnRH receptors in the pituitary gland leads to increased secretion of luteinizing hormone (LH) and follicle-stimulating hormone (FSH) [24]. This process transiently increases testosterone production, known as a “testosterone flare” [24-26]. However, negative feedback between testosterone and GnRH receptors causes the downregulation and desensitization of GnRH receptors to androgen signaling [1, 24], leading to eventual depletion of androgens in the system. On the other hand, GnRH antagonists directly disrupt the signaling between the hypothalamus and pituitary. GnRH antagonists bind to GnRH receptors, inhibiting the release of LH and FSH [24]. This achieves the same effect as castration levels of testosterone without the potentially unfavorable “testosterone flare.” However, this absence of “testosterone flare” may be counterbalanced when the drugs are discontinued. One study indicates that testosterone recovery upon discontinuation of GnRH modulators was significantly slower among patients treated with GnRH agonists compared to antagonists, indicating more sustained effects of androgen suppression with GnRH agonists [27••].

### Androgen Receptor Antagonists

While GnRH agonists and antagonists exert their effects on the HPG axis, AR antagonists exert their effects on the AR of prostate cancer cells. Currently, there are two generations of AR antagonists that differ slightly in mechanism, but both inhibit testosterone-induced AR nuclear translocation and resultant transcription cascade, preventing prostate cancer cell growth [28]. First-generation AR antagonists (e.g., bicalutamide, flutamide) bind directly to the AR, preventing subsequent binding of testosterone/DHT [28]. As a result, the AR remains in the cytosol without nuclear translocation, inhibiting the necessary transcription cascade for promoting cancer cell proliferation and ultimately triggering cellular apoptosis [1, 3]. The second-generation AR antagonists (e.g., enzalutamide and apalutamide) go one step further [28]. In addition to binding to the AR and thereby preventing its binding to androgens, it prevents the AR translocation from the cytoplasm to the nucleus, impairs transcription co-activator recruitment, and restrains AR-DNA binding [28]. These effects work synergistically to augment the inhibitory effects of AR actions, ultimately causing cancer regression.

### Androgen Synthesis Inhibitors

While the previously mentioned ADTs have focused on inhibiting the androgen signal cascade at the HPG axis or prostate cell level, androgen synthesis inhibitors focus on the androgen itself [1, 29]. Androgen synthesis inhibitors exert their effects on the adrenal gland and halt the production of androgens such as testosterone [29]. Inhibiting the production of androgens prevents androgen-driven PCa proliferation. An example of an androgen synthase inhibitor is the CYP17 inhibitor (e.g., abiraterone). CYP17 is an enzyme involved in the conversion of precursor steroids to testosterone and DHT [28]. Thus, CYP17 inhibitors act by inhibiting this conversion and lower serum testosterone and DHT to castration levels, especially when used concurrently with GnRH modulators. This process ultimately leads to cessation of cancer growth.

### Orchiectomy

Bilateral orchiectomy is the surgical castration form of ADT [2]. This procedure results in the removal of the testes, eliminating the production of androgens [29] and thereby halting the androgen signaling cascade. Before the development of pharmacological hormone therapy, bilateral orchiectomy was the traditional “gold standard’ for ADT because it was a relatively rapid and inexpensive modality to decrease serum testosterone levels [2, 30]. With recent advances in pharmacological interventions to achieve castrations, however, chemical castration has become a more favored choice for ADT due to its less invasive nature [2].

## Cardiovascular Effects of Different Types of ADT

Many studies have demonstrated that ADT improves the prognosis of PCa by increasing metastasis-free or survival period in patients [31]. Despite its therapeutic benefits, there is growing concern regarding the increased risk of CVD in patients receiving ADT. Through the Surveillance, Epidemiology, and End Results (SEER)-Medicare study, Keating et al. first reported that ADT, particularly GnRH agonist treatment, is associated with increased risk of CVD [32]. Saigal et al. found that PCa patients who received ADT for at least 1 year had a 20% greater risk of significant cardiovascular morbidity than men who did not receive ADT [33]. These results have sparked considerable interest and discussion regarding the possible association between ADT and increased cardiovascular risk. In 2010, the American Heart Association, American Cancer Society, and American Urological Association evaluated the available data and stated that there is substantial evidence to delineate a relationship between ADT and cardiovascular risk [34]. It is important to note, however, that there remains considerable controversy surrounding the association between ADT and CVD. A number of observational and randomized controlled trials (RCT) have found no link between ADT and cardiovascular events. Punnen et al. reported no significant difference in cardiovascular mortality between those who received ADT and those who did not [35]. Interestingly, a multivariable analysis by Kim et al. demonstrated that ADT actually reduced the risk of CVD (HR 0.89; 95% CI 0.0846–0.936; *p* < 0.0001) [36]. According to Butler et al., the use of ADT plus radiation therapy (RT), compared to RT alone, was not linked with an elevated risk of CVD, even among men with preexisting comorbidities and CVD [37•].

Inconsistent data in the literature may be attributed to heterogeneity in study design, comparisons to age-matched groups rather than PCa patients not receiving ADT, and omission of CV risk assessment at baseline. In particular, accumulating data suggest that those with preexisting CVD or CV risk factors may be at a heightened risk for developing cardiovascular complications following ADT [2]. Therefore, it is critical to characterize cardiovascular complications of different types of ADT to identify and manage cardiovascular risks and complications in PCa patients, especially those of advanced age and with history of CVD. This section aims to provide a comprehensive overview of cardiovascular adverse effects induced by various forms of ADT (Table 2, Fig. 1B).

**Table 2:** Cardiovascular adverse effects associated with androgen deprivation therapy

First author, year	Study type	Pts enrolled (*n*)	Types of treatment	Time frame	Results
General cardiovascular adverse effects associated with ADT					
Keating 2006	Observational	73,196	GnRH agonist Non-ADT	Patients diagnosed from 1992 to 1999, observed through 2001	Increased risk of DM, MI, CAD, MI, and SCD in GnRH agonist group vs. non-therapy group DM: HR, 1.44; *p* < 0.001 CHD: HR, 1.16, *p* < 0.001 MI: HR, 1.11; *p* = 0.03 SCD: HR, 1.34; *p* < 0.004
Saigal 2007	Retrospective	22,816	ADT group Non-ADT group	Data from 1992 to 1996 population-based registry	Patients receiving ADT had 20% higher risk of CV complications compared to similar men who did not receive ADT. (*p* < 0.001)
Zapatero 2016	Phase III RCT (DART 01/05)	355	STAD (4 mo) + HDRT LTAD (24 mo)	Clinical data from 2005 to 2010	Long-term ADT is significantly correlated with a higher incidence of cardiovascular events (HR, 2.090; 95% CI, 1.170–3.720; *p* = 0.012)
No significant CV adverse effects associated with ADT					
Punnen 2011	Post hoc analysis	7248	Local only, primary ADT monotherapy, local treatment + ADT, watchful waiting/active surveillance	Data from 1995 to 2007	Patients treated with WW/AS had a greater risk of cardiovascular morality than patients treated with primary ADT
Nguyen 2011	Meta-analysis of RCTs	4141	GnRH agonist Control Group with no ADT	Data from 1966 to 2011	The incidence of CV deaths between ADT group and control group was not significantly different (255/2200 vs. 252/1941 events, respectively)
Kim 2021	Cohort	131,189	ADT group (*n* = 31,579) Non-ADT Group (*n* = 99,610)	Data from 2008 to 2017	Univariable analysis showed that ADT increased CVD risk, but multivariable analysis showed that ADT independently reduced these risks (HR 0.890; 95% CI, 0.846–0.936; *p* < 0.0001)
Butler 2021	Secondary analysis	1463	ADT plus RT RT alone	Data from 1993 to 2001	Risk of 5-year CV mortality between the ADT plus RT group and RT group alone was not significantly different (2.3% vs. 3.3%, respectively; HR, 0.69; 95% CI, 0.38–1.24; *p* = 0.21)
Voog 2021	Retrospective analysis	1979	RT plus ADT RT alone	Clinical data from 1994 to 2001	Short-course ADT improved overall survival without increased risk of cardiovascular mortality (HR, 1.07; 95 % CI, 0.81–1.42; *p* = 0.62)
Comparison: Cardiovascular adverse effects in GnRH agonist vs. GnRH antagonist					
Albertson 2014	Post hoc analysis	2328	GnRH agonist GnRH antagonist	Data from six phase 3 RCTs between 2005 and 2012	At one year, risk of cardiac events in men treated with GnRH antagonist was significantly lower than those treated with GnRH agonists (HR, 0.44; 95% CI, 0.26–0.74; *p* = 0.0002)
Margel 2019	RCT phase II trial	80	GnRH agonist (*n* = 39) GnRH antagonist (*n* = 41)	1 year	20% of GnRH agonist patients experienced significant cardiovascular and cerebrovascular events compared to 3% of those on GnRH antagonist (*p* = 0.013)
Shore 2020	RCT phase III trial	930	GnRH agonist (*n* = 622) GnRH antagonist (*n* = 208)	48 weeks	GnRH antagonist had a 54% decreased risk of CV adverse effects compared to GnRH agonists (HR, 0.46; 95% CI, 0.24–0.88)
Lifshitz 2021	Post hoc analysis	80	GnRH agonist (*n* = 39) GnRH antagonist (*n* = 41)	1 year	Proteomic analysis showed that GnRH antagonists had a direct protective effect on plaque stability compared to GnRH agonists that had a deleterious effect
Abufaraj 2021	Meta-analysis of RCTs	2632	GnRH agonist (*n* = 986) GnRH antagonist (*n* = 1646)	Data from 2019 to 2020	GnRH antagonist was associated with fewer CV events (RR: 0.52; 95% CI, 0.34–0.80) and lower overall mortality rates (RR: 0.48; 95% CI, 0.26–0.90, *p* = 0.02) compared to GnRH agonists
Lopes 2021	RCT	545	GnRH agonist GnRH antagonist 1:1 ratio	1 year	No significant differences in major CV adverse outcomes between the two research arms *Trial was terminated prematurely
Cardiovascular adverse effects in AR inhibitors and CYP17 inhibitors					
Melong 2017	Experimental	n/a	AR inhibitor	24 hr drug treatment with 13 μM enzalutamide	In zebrafish embryos, enzalutamide induced bradycardia, and increased mortality in a dose-dependent manner
Moreira 2017	Meta-analysis of RCTs	5,183	Abiraterone-Prednisone Placebo-prednisone Enzalutamide Placebo	Data from 1966 to 2015	Abiraterone was associated with increased risk of all-grade (RR, 1.28; 95% CI, 1.06–1.55) and grade ≥ 3 cardiovascular events (RR, 1.76; 95% CI, 1.12–2.75) while enzalutamide was not
Salem 2019	Translational study	n/a	ADT Non-ADT	Data from 1967 to 2018	AR inhibitor enzalutamide inhibited delayed rectifier potassium current and enhanced late sodium current, a potential explanation for QT prolongation and TdP risk seen in ADT patients. Enzalutamide was also associated with more deaths than other types of ADT (5430/31,896 [17%]; *p* < 0.0001)
Bretagne 2020	Post hoc pharmacovigilance analysis	n/a	CYP17 inhibitor Other ADT therapies	Mean time to onset of AT and HF ~ 4–5 months	Abiraterone increased risk of atrial tachyarrhythmias (AT), HF, hypokalemia, hypertension, and edema, all of which are consistent with its hyper-mineralocorticoid effects. Mean time to AT and HF was shorter on abiraterone vs. other ADT (5430/31,896 [17%]; *p* < 0.0001)
Higano 2020	Systematic review	n/a	GnRH agonist GnRH antagonist AR-targeted agents + ADT ADT + placebo	Data from 1941 to 2020	AR-targeted agents used with ADT has shown increased hypertension and/or CV risk compared to the ADT + placebo groups in several phase 3 trials
Fizazi 2022	RCT phase III trial	1173	Standard of care (SoC) (*n* = 296) SoC + RT (*n* = 293) SoC + CYP17 inhibitor (*n* = 292) SoC + RT + CYP17 inhibitor (*n* = 291)	Enrollment 2013–2018, median follow-up 3.5 years	CYP17 inhibitor abiraterone + ADT + docetaxel improved progression-free and overall survival in mCRPC patients with modest increase in hypertension. Patients who received abiraterone had longer radiographic progression-free survival (HR, 0.54; 99.9% CI, 0.41–0.71; *p* < 0.0001) and overall survival (0.82; 95.1% CI, 0.69–0.98; *p* = 0.030) than patients who did not receive abiraterone
Cardiovascular adverse effects in orchiectomy					
Keating 2006	Observational	73,196	Orchiectomy	Patients diagnosed from 1992 to 1999, observed through 2001	Increased risk of DM (HR, 1.34; *p* < 0.001) and no associated CV events (*p* > 0.20) in the orchiectomy group
Teoh 2015	Post hoc analysis	684	Bilateral orchiectomy (*n* = 387) GnRH agonist (*n* = 297)	Data from 2000 to 2009	Surgical castration by bilateral orchiectomy increased risk of acute MI and ischemic stroke in Chinese PCa patients
Chen 2017	Post hoc analysis	14,715	Bilateral orchiectomy (*n* = 3578) GnRH agonist (*n* = 11,137)	Data from 1997 to 2011	Orchiectomy is linked to an increased risk of CV events, particularly in older patients and in those with CV comorbidities. CV events include MI, HF, SCD, and mortality)

Abbreviation: *AR*, androgen receptor; *ADT*, androgen deprivation therapy; *CAD*, coronary artery disease; *CI*, confidence interval; *CVD*, cardiovascular death; *CVM*, cardiovascular mortality; *CrVD*, cerebrovascular death; *DM*, diabetes mellitus; *HF*, heart failure; *HR*, hazard ratio; *LTAD*, long-term androgen deprivation; *MI*, myocardial infarction; *RR*, risk ratio; *RT*, radiation therapy; *SCD*, sudden cardiac death; *STAD*, short-term androgen deprivation

### Cardiovascular Adverse Effects: GnRH Agonists vs. Antagonists

While it is generally accepted that ADT may increase the risk of CVD, the relative CVD risk among ADT types differs and must be appreciated in order to develop a medically optimal and individualized management plan for PCa patients. Currently, injectable GnRH agonists (e.g., leuprolide) are the standard agents for achieving castrate-level testosterone for PCa [27••]. However, several studies have noted that GnRH agonists have increased CVD risk profile compared to GnRH antagonists (e.g., degarelix). In the SEER-Medicare observational study, men receiving GnRH agonists had a significantly increased risk of incident diabetes (adjusted HR, 1.44; *p* < 0.001), coronary heart disease (adjusted HR, 1.16; *p* < 0.001), and myocardial infarction (adjusted HR, 1.11; *p* < 0.03) [32] compared to men not receiving treatment. Similarly, a MEDLINE search reviewing the literature from 1986 to 2008 found an increased risk for CVD within months of beginning ADT administered by GnRH agonists (e.g., goserelin, histrelin, and leuprolide) [38]. In 2010, the US Food and Administration (FDA) reviewed data from several studies and issued new safety labels on GnRH agonists pertaining to increased risk of diabetes, heart attack, sudden cardiac death, and stroke. Several studies published over the following decade continued to show an elevated risk of CV events in PCa patients treated with GnRH agonists compared to patients treated with GnRH antagonists or no treatment. Recently, a phase II, prospective, open-label study randomized 80 patients to receive GnRH agonists or antagonists for 1 year [39•]. While no differences in endothelial function (primary endpoint) were noted between the two groups, significantly more cardiovascular events (secondary endpoint) were reported in the GnRH agonist arm compared to the GnRH antagonist arm (20% vs. 3%, respectively, *p* = 0.013). At 12 months, using a GnRH antagonist conferred an absolute risk reduction in major cardiovascular and cerebrovascular events by 18.1% (95% CI 4.6–31.3, *p* = 0.032); CVD in this study was defined as myocardial infarction (MI), cerebrovascular events, death, percutaneous angioplasty, or hospitalizations due to cardiac events. In the landmark phase III HERO trial, GnRH antagonist relugolix was found to be superior to GnRH agonist leuprolide. Relugolix adequately and rapidly suppressed testosterone levels in PCa patients without clinical “flare” and with a 54% decreased risk of significant adverse cardiovascular events (HR, 0.46; 95% CI, 0.24–0.88) [27, 40].

### Cardiovascular Adverse Effects of AR Inhibitors and CYP17 Inhibitors

AR inhibitors (e.g., enzalutamide) and CYP17 inhibitors (e.g., abiraterone) are newer-generation androgen deprivation therapies used for metastatic castration-resistant prostate cancer (mCRPC). As discussed in the earlier section, these drugs target the androgen receptor or its activation, and both have been shown to be clinically effective, particularly in high-risk prostate cancer patients [41]. While enzalutamide and abiraterone both significantly increase survival benefits, they are also associated with various adverse events such as fatigue, back pain, hypertension, hypokalemia, and edema [42]. CV risk profiles of these therapies, however, are yet to be established and further investigation is required to confirm their efficacy and safety. A translational study from the international pharmacovigilance database (VigiBase) found that enzalutamide was associated with the highest rate of death (*p* < 0.0001) when compared to other ADTs, including degarelix, abiraterone, and leuprorelin [43], due in part to its association with acquired long QT syndrome (aLQTS), torsades de pointe, and sudden death. These results are supported by in vivo and in vitro studies, which demonstrated enzalutamide’s role in prolonging the cardiac action potential by inhibiting delayed rectifier potassium current and chronically enhancing late sodium current [44, 30]. Mean times to AT and HF onset were shorter with use of abiraterone (5.2 ± 0.8 and 4.5 ± 0.6 months, respectively) compared to other ADTs (13.3 ± 3.2 and 9.2 ± 1.1 months, respectively) (both *p* < 0.05). Hypokalemia was also seen more frequently with the use of abiraterone (6/21 [29%]) than in other ADT (7/163 [4%]; *p* < 0.0001) [43]. In another observational study through VigiBase, one population-based cohort study found that CVD risk was increased in men on abiraterone and enzalutamide (HR, 1.19; 95% CI: 1.03–1.38) and (HR 1.10; 95% CI: 1.01–1.120), respectively [45]. Other RCTs have found an increased risk of CVD in just abiraterone only, whereas enzalutamide was only associated with an increased risk of hypertension [46].

### Cardiovascular Adverse Effects of Orchiectomy

Though the utilization of surgical castration has declined over time [47], multiple studies have deemed orchiectomy to be superior in reducing testosterone to castrate levels and overall survival [48]. Some studies even show that bilateral orchiectomy is associated with reduced risk of peripheral arterial disease, CV events, and diabetes mellitus [49]. Despite improving disease control in PCa, other studies have found a positive association between orchiectomy and cardiovascular complications. Compared with GnRH agonists, bilateral orchiectomy was shown to increase risk in CV events, especially in patients of older age and with history of CV comorbidities [50]. Similarly, Teoh and colleagues noted orchiectomy to be a significant risk factor for MI and ischemic stroke [51]. These studies are limited, however, due to their retrospective nature and lack of standardized protocol. Other factors such as treatment dose and duration may also affect the accuracy of study results. Additionally, because medical castration is preferentially utilized over surgical castration contemporarily, there seems to be a lack of data on the cardiovascular risk profile of orchiectomy. Further prospective studies investigating the safety of orchiectomy are warranted for clinicians to consider the risk–benefit ratio of all ADT treatment options when managing PCa.

## Cardiovascular Management Considerations when Using ADTs

The first step when caring for prostate cancer patients being considered for ADT is to be aware of the potential cardiovascular complications of ADT as described above and to comprehensively assess patients’ baseline cardiovascular health, especially regarding their preexisting CVD and/or risk factors. Before initiating an ADT, if patients have prior history of CVD or multiple CV risk factors, the care team may consider referral to cardiology or cardio-oncology for further assessment and CV optimization, as well as for multidisciplinary discussion regarding the risks and benefits of different types of ADT. Second, during the course of ADT, it is recommended that the care team focuses on CVD prevention by managing CV risk factors; for instance, patients should be encouraged to follow American Heart Association (AHA)’s 8 life’s essential key measures for improving and maintaining cardiovascular health, which include to (1) eat better, (2) be more active, (3) quit tobacco, (4) get healthy sleep, (5) manage weight, (6) control cholesterol, (7) manage blood sugar, and (8) manage blood pressure (https://www.heart.org/en/healthy-living/healthy-lifestyle/lifes-essential-8). While there are no specific CVD monitoring guidelines for patients on ADT, the care team should have a lower threshold for monitoring and treating the patients on ADT for possible CVD given the heightened risk of CV events compared to the general population. Otherwise, the treatment for adverse cardiovascular events such as MI or HF should follow standard guidelines. Finally, patient involvement in treatment decisions as well as patient education regarding potential adverse CV effects during the course of ADT would be important in the overall care given the often long-term nature of ADT.

## Conclusion

The use of ADT has been instrumental in reducing mortality rates in PCa patients. The mechanism of action for various types of ADT ties into a negative feedback loop regulated by the endocrine system, which is tightly coordinated by various essential modulators designed to either downregulate or overstimulate hormone production, which has been effective in slowing cancer growth. However, the adverse cardiovascular implication associated with long-term ADT use is profound and needs to be addressed to ensure that risk is at a minimum. The potential cardioprotective role of androgens regarding heart health has only partially been elucidated, and more research needs to be conducted to evaluate how/why ADTs lead to CVD. For instance, future studies could potentially perform gene expression and bioinformatic analysis to gain an in-depth understanding of the plethora of genes that are upregulated or downregulated in the cardiovascular system modulated by the different types of ADT. This will allow healthcare professionals to better understand how each drug affects individual patients differently, thus creating a platform for personalized medicine. This customized approach will ultimately serve as the future of cancer therapy and the management of CV complications.
